# Emission Rate of Particulate Matter and Its Removal Efficiency by Precipitators in Under-Fired Charbroiling Restaurants

**DOI:** 10.1100/tsw.2011.103

**Published:** 2011-05-26

**Authors:** Jun-Bok Lee, Ki-Hyun Kim, Heung-Joo Kim, Seog-Ju Cho, Kweon Jung, Shin-Do Kim

**Affiliations:** ^1^ Department of Environmental Engineering, University of Seoul, Seoul, Republic of Korea; ^2^ Department of Environment and Energy, Sejong University, Seoul, Republic of Korea; ^3^ Seoul Metropolitan Government Institute of Public Health and Environment, Seoul, Republic of Korea

**Keywords:** emission rates, UFC restaurant, emission factor, PM, precipitator

## Abstract

In order to explore the potent role of meat cooking processes as the emission sources of particulate matter (PM), emission rates and the associated removal efficiency by precipitators were estimated based on the on-site measurements made at five under-fired charbroiling (UFC) restaurants. The emission patterns of PM for these five restaurants were compared after having been sorted into the main meat types used for cooking: beef (B), chicken (C), intestines (I), and pork (P: two sites). The mass concentrations (μg m^-3^) of three PM fractions (PM_2.5_/PM_10_/TSP) measured from these restaurants were 15,510/15,701/17,175 (C); 8,525/10,760/12,676 (B); 11,027/13,249/13,488 (P); and 22,409/22,412/22,414 (I). Emission factors (g kg^-1^) for those PM fractions were also estimated as 3.23/4.08/4.80 (B), 3.07/3.82/3.87 (P), 8.12/8.22/8.99 (C), and 6.59/6.59/6.59 (I). If the annual emission rate of PM_10_ is extrapolated by combining its emission factor, population, activity factor, etc., it is estimated as 500 ton year^-1^, which corresponds to 2.4% of the PM_10_ budget in Seoul, Korea. Removal efficiencies of PM_10_ via precipitators, such as an electrostatic precipitator (ESP), bag filter (BF), and the combination system (ESP + catalyst), installed in those UFC restaurants ranged between 54.76 and 98.98%. The removal efficiency of PM by this control system was the least effective for particles with <0.4 μm, although those in the range of 0.4–10 μm were the most effective.

### INTRODUCTION

Certain cooking processes are now known to release undesirable pollutants into the atmosphere. In this respect, meat cooking processes are suspected as one of the major emission sources of particulate matter (PM) and associated compounds, e.g., secondary organic aerosols (SOA), organic carbon (OC), elemental carbon (EC), etc.[1,2]. The potent role of under-fired charbroiling (UFC) restaurants has been, in fact, recognized as the greatest emitter of PM and volatile organic compounds (VOCs)[3]. Schauer et al.[1] showed that emissions from meat charbroiling and frying can account for about 20% of all fine organic

PM or approximately 7% of the total fine particle mass released into the atmosphere of Los Angeles, U.S. In recognition of the prevalence of the meat cooking process, some regulations for its emissions were implemented, including San Joaquin Valley Unified Air Pollution Control District (SJVUAPCD) Rule 4692[4], South Coast Air Quality Management District (SCAQMD) Rule 1138[5], and Bay Area Air Quality Management District (BAAQMD) Regulation 6, Rule 2[6].

Emission inventories are regarded as an indispensable tool for environmental measures designed to prevent air pollution. In creating emission inventories of major pollutants (PM, $PM_{10}$, $PM_{2.5}$, VOCs, PAHs, CO, NOx, and $SO_2$), the U.S. Environmental Protection Agency (EPA)[7] has estimated emission factors and emission rates at UFC restaurants on a per-year basis. In the case of Korea, national and/or local emission inventories are not yet established with respect to UFC restaurants. There are only a limited number of studies that have attempted to measure emissions from restaurants or households[8,9,10,11,12,13,14,15]. As a result, the emission database for this category relies on several reports made in the few earlier studies.

The reduction of emission sources is regarded as an important environmental measure to prevent air pollution. One of the most efficient approaches for this reduction is to operate proper control equipment. Precipitators, such as an electrostatic precipitator (ESP), bag filter (BF), and some combined systems (e.g., ESP + catalyst), are available for the removal of PM. However, the use of such devices as precipitators in restaurants is as yet infrequent because they are not yet enforced via legislative regulations in Korea. To date, the removal efficiency of such precipitators has not been thoroughly investigated in Korea. To learn more about the source-control relationship of airborne pollutants, we estimated emission rates of PM released from UFC restaurants and evaluated their removal efficiency against diverse control equipment in practice. To this end, we conducted a series of field campaigns to measure PM emissions from real-life operations of UFC processes. This study also reports the results of our investigation to examine the feasibility of PM removal facilities currently employed at the target UFC restaurants.

### MATERIAL AND METHODS

#### The Selection of Target Restaurants

In order to select the target restaurants for study, we conducted an initial survey of all UFC restaurants registered in the city administrative office of Seoul as of 2008. The results showed a total of 10,103 UFC restaurants in its boundary, among which 1,649 can be classified as large-scale UFC restaurants (i.e., the area exceeding 100 $m^2$). The fuels used in those large-size restaurants consisted of charcoal (960 sites), liquefied petroleum gas (669), wood (11), briquette (7), and straw (2). Out of those 1,649 restaurants, 14 UFC restaurants were identified as having pollution control systems. Table 1 shows the list of five UFC restaurants selected randomly (out of 14) for this investigation that are equipped with precipitators such as an ESP (three sites; 4,000–10,000 $m^3\ h^{-1}$), ESP + catalyst (one site; 3,000 $m^3\ h^{-1}$), and BF (one site; 4,800 $m^3\ h^{-1}$). All of the five selected restaurants used Korean charcoal as the main fuel. The main meat types cooked for charbroiling at these restaurants were chicken (C) (one site), beef (B) (one site), pork (P) (two sites), and intestines (I) (one site). The collection of PM precipitators was made through the hood and duct system. The hood and duct systems at each restaurant were installed to cover as many as 12–30 table sets for each group of dining teams (up to four persons for each table).

#### Sampling and Analysis

In order to measure the concentrations of PM and to develop its emission factors, the procedures described in the EPA method 201A were applied with a slight modification for this study. The EPA method 201A is the in-stack measurement of PM equal to or less than an aerodynamic diameter of nominally

**TABLE 1**
**Five Restaurants Investigated in this Study[a]**

| Restaurant Symbol | Main Meat Types Cooked | Area ($m^2$) | No. of Dining Tables[b] | Control/Precipitator (capacity in $m^3$ $h^{-1}$) |
|---|---|---|---|---|
| A | C | 106 | 15 | ESP (4,000) |
| B | B | 165 | 14 | ESP (4,000) |
| C | P | 785 | 80 (12)[c] | ESP + catalyst (3,000) |
| D | I | 300 | 30 | ESP (10,000) |
| E | P | 205 | 20 | BF (4,800) |

[a] Fuel type used in all restaurants: charcoal.

[b] Total number of dining tables.

[c] Number in the parentheses denotes those tables connected to hood and duct system.

10 ($PM_{10}$) and 2.5 μm ($PM_{2.5}$), along with total suspended particle (TSP). To comply with the sampling train of method 201A, the $PM_{2.5}$ cyclone is inserted between the $PM_{10}$ cyclone and the filter. The $PM_{10}$ cyclone collects the fraction greater than $PM_{10}$, while the $PM_{2.5}$ cyclone collects the PM fractions between 2.5 and 10 μm. Thus, TSP is collected on the front side of the $PM_{10}$ cyclone, while $PM_{10}$ and $PM_{2.5}$ are collected on the in-line sampling cylinder (between the two cyclones) and on the in-line filter (Quartz), respectively. For the isokinetic collection of in-stack samples, isokinetic coefficients were maintained at a constant value of 95–110%, which is recommended by the Korean standard testing method. During our field campaigns, the collection of the PM samples from each facility was carried out one time between 19:00 and 21:00 in light of intensive cooking activities in that time band. More details for sampling conditions in facilities, such as sampling date, sampling time, suction flow rate, etc., are described in Table 2. Filters were placed in a weighing chamber over 24 h, both before and after sampling, and weighted with a microbalance (UMX2, Mettler Toledo Inc., SWI).

**TABLE 2**
**Sampling Conditions of Three PM Fractions in Each Facility**

| Item | A | B | C | D | E |
|---|---|---|---|---|---|
| Sampling date[a] | 9/18 | 10/21 | 10/27 | 10/29 | 11/5 |
| Velocity (m $sec^{-1}$) | 6.40 | 4.06 | 4.13 | 5.23 | 6.72 |
| Flow rate (Q: $m^3$ $sec^{-1}$)[b] | 1.6 | 0.8 | 0.4 | 1.5 | 1.3 |
| Wt (kg) of meat | 5.50 | 3.80 | 3.50 | 9.18 | 4.55 |
| Suction flow rate ($m^3$ $sec^{-1}$)[c] | 384 | 206 | 120 | 180 | 234 |

[a] Month/date in 2009.

[b] Sampling time = 30 min.

[c] Suction flow rate for dining tables connected to hood and duct system.

The removal efficiency of PM is affected by several governing factors, including concentration, size distribution, etc. Therefore, removal efficiencies can be estimated in relation to the particle size distribution. To estimate the removal efficiency of PM, the optical aerosol spectrometer (OAS: Grimm 1.109, Grimm Aerosol Technique, Germany) equipped with isokinetic sampling probes (Grimm 1.152,

Grimm Aerosol Technique, Germany) was employed to count PM levels, both up- and downstream of the precipitator. This OAS measures the particle count with 31 channels (diameter range: 0.25–32 μm) on a real-time basis at every 6 sec. As there is no international standard for the calibration of the OAS, its calibration was made by referring to ISO 12103 A3 (intercomparison method). As part of the calibration, a statistical comparison was made between this analyzer and a precalculated traceable reference unit (mother unit). The precalculation step was carried out with a combination of different monodisperse Poly Styrene Latex traceable standard particles (Duke Scientific Inc., U.S.) and standard equipment of LAS-X aerosol particle counter (PMS Inc., U.S.). The statistical comparison is then made against mean values of the particle count at each channel. This calibration is repeated automatically by a computer software that adjusts the data with the accuracy of ±2%.

### Theoretic Basis

Emission factor (EF) is typically defined as the amount of a model pollutant emitted per unit mass of fuel burned[16]. It is often expressed in terms of mass-based EF with a mass/mass ratio unit (e.g., g $kg^{-1}$). The concept of EF is generally used for the calculation of emission inventory. In this study, we adopted the procedures of Lee et al.[17] to apply mass-based EF.

$$Emission\ \ factor\,(g\ kg^{-1}\ meat) = \frac{C(ug\ m^{-3}) \times Q(m^3 s^{-1}) \times sampling\ time(\min) \times 60(s\ \min^{-1})}{Wt(kg) \times 10^6 (ug\ g^{-1})}$$

where C and Q are the concentration of PM and the flow rate of air (passing through the duct), respectively. The latter term is calculated by the equation of V (velocity) × A (area of inlet duct) over 30 min of sampling time. Wt is the weight of charbroiled meat.

The emission rates for each meat type were then computed by the following equations:

$$Emission\ \ rate(ton\ yr^{-1}) = k \times EF(g\ kg^{-1}) \times CR(kg\ person^{-1}\ yr^{-1}) \times P(person) \times 10^{-6} (ton\ g^{-1})$$

where k is activity factor, EF is emission factor of PM, CR is consumption rate of each meat type, and P is the population. The individual parameters, such as k, CR, and P, were derived for the entire city of Seoul as of 2008[18,19,20].

Removal efficiencies of PM by all the precipitators were also evaluated as follows:

$$Removal\ efficiency\ (\%) = \frac{PM(i) - PM(o)}{PM(i)} \times 100$$

where PM(i) and PM(o) denote the particle number concentration (counts) for up- (inlet) and downstream (outlet), respectively.

## RESULTS AND DISCUSSION

### Mass Concentrations of PM

Table 3 shows the mass concentrations of all PM fractions measured at each of the five UFC restaurants. The concentrations (μg $m^{-3}$) of three particle fractions ($PM_{2.5}$, $PM_{10}$, and TSP) in each restaurant were (1) 15,510; 15,701; and 17,175 (C); (2) 8,525; 10,760; and 12,676 (B); (3) 5,168; 7,084; and 7,084 (P1); (4) 16,886; 19,413; and 19,891 (P2); and (5) 22,409; 22,412; and 22,414 (I). The results of this simple comparison

**TABLE 3**

**Mass Concentrations (μg $m^{-3}$) of All Three PM Fractions ($PM_{2.5}$, $PM_{10}$, and TSP) at Each Restaurant and Their Derivative Ratios of $PM_{2.5}/PM_{10}$ and $PM_{10}$/TSP[a]**

| PM Fractions and Ratios | A | B | C | D | E |
|---|---|---|---|---|---|
| $PM_{2.5}$ | 15,510 | 8,525 | 16,886 | 22,409 | 5,168 |
| $PM_{10}$ | 15,701 | 10,760 | 19,413 | 22,412 | 7,084 |
| TSP | 17,175 | 12,676 | 19,891 | 22,414 | 7,084 |
| $PM_{2.5}/PM_{10}$ | 0.99 | 0.79 | 0.87 | 1.00 | 0.73 |
| $PM_{10}$/TSP | 0.91 | 0.85 | 0.98 | 1.00 | 1.00 |

[a] Capital letters A thorough E were assigned to five arbitrarily allocated UFC restaurants, as shown in Table 1.

indicate that the UFC restaurant cooking with large intestine was the most distinct contributor of all particle fractions ($PM_{2.5}$, $PM_{10}$, and TSP) in terms of mass concentrations.

In order to assess the effect of natural ventilation on PM concentration, the ratios between the indoor and outdoor PM concentrations had to be evaluated. These values indicate how well the building ventilation system disperses the indoor-generated PM and thus affects the outdoor PM concentration[21]. To this end, the outdoor concentrations of PM in this study were represented by those samples collected separately through the hood and duct system. Table 3 also shows that the ratios of $PM_{2.5}/PM_{10}$ and $PM_{10}$/TSP measured at UFC restaurants were 0.73–1.00 and 0.85–1.00. (Note that the same ratios derived from ambient air were 0.48 and 0.66, respectively, if one refers to the annual average concentrations [μg $m^{-3}$] of $PM_{2.5}$ [26], $PM_{10}$ [54], and TSP [82] measured at 25 air quality monitoring stations in Seoul, 2009.) This computation thus indicates that the $PM_{2.5}$ should be the predominant component of PM emissions at all target restaurants.

In a previous report by the EPA[9], particle size distribution was found to be quite comparable between different meat types, while $PM_{2.5}$ and $PM_{10}$ constituted 80 and 20%, respectively. Welch and Norbeck[22] also confirmed that a majority fraction of particles was $PM_{2.5}$ on the basis of the measurements made at 18 commercial cooking restaurants. Furthermore, Lee et al.[17] showed that the accumulated mass concentrations of $PM_{10}$ from the UFC process were 92.2–99.5%, with their dominant fraction in particle diameter range of 2.0–2.5 μm. Park et al.[23] also reported that the median diameter of PM released from meat cooking was 2.4 μm Therefore, it is reasonable to infer that PM released from UFC restaurants should primarily consist of $PM_{2.5}$.

### Emission Rates

#### *Emission Factors*

We developed emission factors of PM based on *in situ* data collected during real-life operations at all selected restaurants. The results of our measurements made at the UFC restaurants were then used for the estimation of emission inventory of PM. Table 2 summarizes the sampling conditions of PM at each restaurant, e.g., measured velocity, flow rate, sampling time, and weight of meats charbroiled (as parameters for calculating emission factors). The ranges of air velocity and flow rate at each restaurant were 4.06–6.72 m $sec^{-1}$ and 0.4–1.6 $m^3$ $sec^{-1}$ (1,440–5,760 $m^3$ $h^{-1}$), respectively (Table 2). The suction flow rates for dining tables connected to a hood and duct system were 120–384 $m^3$ $h^{-1}$. From all five restaurants, sampling of PM was made for the duration of 30 min. Information concerning the total quantity of charbroiled meats was obtained from the manager of each restaurant before investigation. The weights of charbroiled meat samples for the test were 3.50–9.18 kg for the five UFC restaurants.

Our emission factors for UFC restaurants using charcoal fuels were compared with those quantified previously in three separate studies (Table 4). All of these emission factors were computed by considering the contribution of both meat and charcoal fractions. However, emission factors of the charcoal were not divided separately, as the relative contribution is trivial under most circumstances. The EPA[9] reported that charcoal did not contribute significantly to PM emission levels. In addition, Lee et al.[17] reported that emission factors of charcoal were 0.01–0.02 ($PM_{2.5}$), 0.01–0.03 ($PM_{10}$), and 0.02–0.05 g $kg^{-1}$ (TSP) on the basis of the experimental results derived by the hood method. The basic concept of this method is to equip a hood and exhaust duct above the cook stove to collect all emissions[16]. In this study, $PM_{2.5}$/$PM_{10}$/TSP emission factors (g $kg^{-1}$) of C, B, P, and I were 8.12/8.22/8.99, 3.23/4.08/4.80, 3.07/3.82/3.87, and 6.59/6.59/6.59, respectively. Relative ordering in emission factors were, in some senses, consistent between B and P across different particle fractions. It is also interesting to note that emission factor values of C were the largest in both particle fractions. In addition, all emission factors developed in this study were low relative to the previous studies (Table 4).

**TABLE 4**
**Comparison of Emission Factor (EF) Estimates of Three Major PM Fractions (in g $kg^{-1}$) between Different Studies[a]**

| Meat Type | PM Fractions | EF | | | |
|---|---|---|---|---|---|
| | | This Study | Pechan (2003) | EPA (1999) | SJVUAPCD (2006) |
| B | $PM_{2.5}$ | 3.23 | 7.1 | — | — |
| | $PM_{10}$ | 4.08 | 7.5 | — | — |
| | TSP | 4.80 | 8.1 | 7.68–7.91 | — |
| P[b] | $PM_{2.5}$ | 3.07 | — | — | 8.98 |
| | $PM_{10}$ | 3.82 | — | — | 9.53 |
| | TSP | 3.87 | — | — | — |
| C | $PM_{2.5}$ | 8.12 | 9.1 | — | 8.98 |
| | $PM_{10}$ | 8.22 | 9.4 | — | 9.53 |
| | TSP | 8.99 | 9.8 | 10.05–10.80 | — |
| I | $PM_{2.5}$ | 6.59 | — | — | — |
| | $PM_{10}$ | 6.59 | — | — | — |
| | TSP | 6.59 | — | — | — |

[a] EF values for each PM fraction released via combustion were derived without distinguishing meat and charcoal.

[b] Mean value was taken from the results of the two restaurants.

It is suspected that all these differences should be ascribed at least partially to different sampling approaches. Note that the basic sampling methods of PM can be classified into two categories: source-level sampling and ambient-level sampling. Source-level sampling, equivalent to the in-stack sampling method (EPA method 201A), was applied in this study. In contrast, ambient-level sampling techniques, employed in previous studies[3,9,24], required a very wide range of space and high costs. The general sampling and analytical principles employed in the analysis of concentration, size, and composition of PM are comparable between the two types of measurement approaches. However, each specific approach differs greatly due to the differences in the prevalent environmental conditions (temperature, concentration, etc.) of sampling[25]. In the case of the PM measurements from stationary sources, source-level sampling is currently recommended in the context of regulatory enforcement, Korean standard testing method[26]. In contrast, ambient-level sampling based on the dilution chamber method is accepted for PM measurement from mobile sources.

### Emission Rates

To estimate emission rates of each PM fraction ($PM_{2.5}$/$PM_{10}$/TSP), the parameters for calculating emission rates were derived from a number of previous reports: (1) consumption ratio (CR)[18], (2) activity factor (k)[18,19], and (3) population (P) of Seoul[20]. However, the CR reported in the survey by the KMTA[18] involved the total amount of meat consumed both in all places of meat consumption (e.g., wholesale market, meat processing factory, restaurants, etc.) and by all cooking methods (e.g., charbroiling, soup, stew, frying, etc.). For an accurate computation of the consumption ratio (CR; kg $person^{-1}$ $year^{-1}$) for each meat type in this study, activity factors (k) for only UFC were taken into consideration. This “k” value was expressed as the proportion (%) representing specific activities. In order to derive the “k” value, we attempted to document both (1) the relative proportion (%) of three meat types (B, C, and P) consumed in restaurants among all the consumption pathways and (2) the ratio of those meats processed via the UFC method among all the cooking approaches. Both activities were applicable to restaurants cooking meat types of B, P, and C. The KMTA[18] reported that the former activities (%) for each of the three meat types were 25.7, 21.7, and 37.0, respectively. In addition, the NIER[19] reported that the latter activities (%) for those three meat types were 61.6, 46.0, and 41.7, respectively, among 1,124 restaurants in Korea. Consequently, their activity factors were calculated as 0.158, 0.100, and 0.154, respectively. Table 5 summarizes activities and the corresponding activity factors.

**TABLE 5**
**Market Share Ratios for Meat Type with the Corresponding Activity Factors**

| | Distribution Ratio (%)[a] | Proportion (%) of UFC Method[b] | Activity Factor (k)[c] |
|---|---|---|---|
| B | 25.7 | 61.6 | 0.158 |
| P | 21.7 | 46.0 | 0.100 |
| C | 37.0 | 41.7 | 0.154 |

[a] Distribution ratios (%) of each type of meat consumed in restaurants to total CR.

[b] Proportions (%) of cooking method by UFC relative to other cooking methods.

[c] Activity factor = (distribution ratio [%] $\times$ proportion [%] of UFC method)/100.

If one assumes that UFC restaurants cook only those three meat types (B,P, and C), their emission rates of $PM_{2.5}$, $PM_{10}$, and TSP are computed as 214, 240, and 260 ton $year^{-1}$, respectively. (Refer to the column of subtotal values in Table 6.) Among 10,103 UFC restaurants in Seoul, the relative proportion of restaurants cooking those three meat types was 48%, as shown in Fig. 1. Hence, through an extrapolation of our experimental results for all the total UFC restaurants in Seoul, their emission rates of the three PM fractions in Seoul are estimated to be 446, 500, and 542 ton $year^{-1}$, respectively.

**TABLE 6**
**Emission Rates of $PM_{2.5}$, $PM_{10}$, and TSP in Seoul, Korea[a]**

| Meat | Consumption Rate (kg person$^{-1}$ year$^{-1}$) | Activity Factor (k) | Emission Rates (ton year$^{-1}$) | | |
|---|---|---|---|---|---|
| | | | $PM_{2.5}$ | $PM_{10}$ | TSP |
| B | 7.5 | 0.158 | 39 | 49 | 58 |
| P | 19.1 | 0.100 | 60 | 74 | 75 |
| C | 9.0 | 0.154 | 115 | 117 | 127 |
| Subtotal | — | — | 214 | 240 | 260 |
| Total[b] | — | — | 446 | 500 | 542 |

[a] Population of Seoul city in 2008 = 10,421,782.

[b] Total = Subtotal/0.48: here, the use of 0.48 is made to represent the proportion of the UFC restaurants cooking the three meat types: C, P, and B.

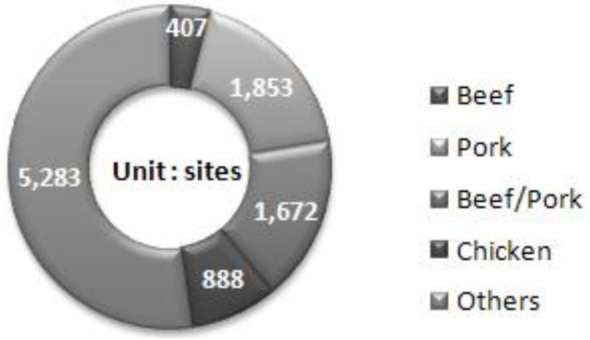

**A**

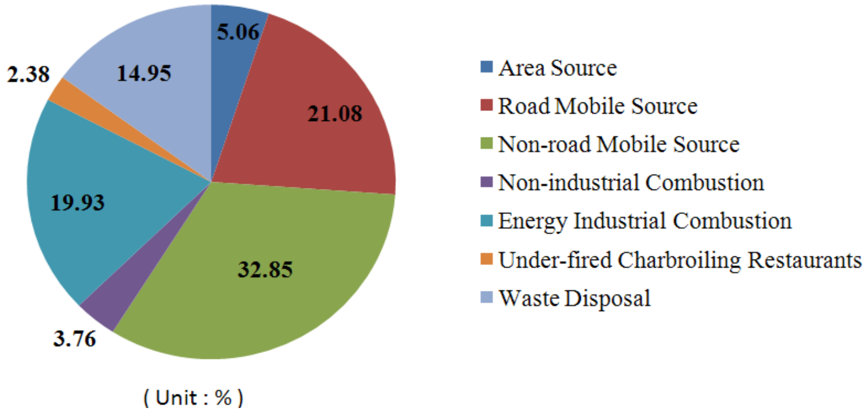

**B**

**FIGURE 1.** Classification of food types for the UFC restaurants and their relative contribution in $PM_{10}$ source apportionment. (A) Classification of UFC restaurants on the basis of the main meat types cooked in Seoul (our initial survey, 2008); (B) relative contribution of UFC restaurants in the total source apportionment of $PM_{10}$ in Seoul (2008).

### *Contribution Ratio of $PM_{10}$*

In order to assess the relative contribution of UFC restaurants to atmospheric PM levels in Seoul (as of 2008), the latest emission ratio data (as of 2007) for all available air pollutants in Seoul were obtained[27]. As shown in Fig. 1B, $PM_{10}$ released from UFC restaurants can contribute to about 2.4% of the ambient $PM_{10}$ levels in Seoul. As $PM_{2.5}$ and TSP are not regulatory air pollutants in Korea, the relative contributions of $PM_{2.5}$ and TSP were not estimated; their source apportionments are not yet established as the component of Korean emission inventory. Considering the $PM_{2.5}/PM_{10}$ ratio (0.73–1.00: this study) and the fraction of $PM_{10}$ (92.2–99.8%) to TSP[17], $PM_{2.5}$ should be the predominant component of airborne particles produced from UFC. Consequently, it is projected that the contribution of $PM_{2.5}$ from UFC restaurants should be much greater to atmospheric PM composition than that of $PM_{10}$.

### Removal Efficiency of PM

The removal efficiencies of each PM fraction were estimated and compared between different types of precipitators (Table 7). Most importantly, the collection efficiency of precipitators is affected very sensitively by particle size distribution. Therefore, the removal efficiencies of $PM_{2.5}$, $PM_{10}$, and TSP (by precipitator) were calculated by integrating the particle count data for each particle size. The integrated particle count data obtained by the OSA were arbitrarily classified into (1) 0.4–2.5 μm (the first stage: 11 channels), (2) 2.5–10 μm (the second stage: eight channels), and (3) 10–32 μm (the third stage: seven channels). These data were assigned to represent $PM_{2.5}$, $PM_{10}$, and TSP, respectively. The removal efficiencies for the three PM fractions ($PM_{2.5}$, $PM_{10}$, and TSP) were then estimated as 54.62–98.98%, 54.76–98.98%, and 89.61–99.96%, respectively. The removal efficiency of bag filter (BF) was the highest of all the matching pairs. In addition, that of the electrostatic precipitator (ESP) had a wide-ranging removal efficiency: 54.62–97.41% ($PM_{2.5}$), 54.76–97.42% ($PM_{10}$), and 89.61–98.32% (TSP). The efficiency of the combined system (ESP + catalyst) for the three fractions was 92.94, 92.95, and 92.95%. Park et al.[23] measured the removal efficiency of the ESP in a manner similar to our study. These authors reported that two-stage ESP collected 89% of TSP in the case of P (their meat type) by the hood method.

**TABLE 7**
**Removal Efficiency of PM According to Precipitator Types**

| Type of Precipitator | Removal Efficiency (%) | | |
|---|---|---|---|
| | $PM_{2.5}$ | $PM_{10}$ | TSP |
| 1. Electrostatic precipitator (ESP) | 54.62–97.41 | 54.76–97.42 | 89.61–98.32 |
| 2. Combination of ESP + catalyst | 92.94 | 92.95 | 92.95 |
| 3. Bag filter (BF) | 98.98 | 98.98 | 99.96 |

Particle count data were integrated in the ranges of (1) 0.4–2.5 μm (the first stage: 11 channels), (2) 2.5–10 μm (the second stage: eight channels), and (3) 10–32 μm (the third stage: seven channels). These data were then applied to the computation of removal efficiencies for each PM fraction of $PM_{2.5}$, $PM_{10}$, and TSP, respectively.

The removal efficiency of both ESP and BF can be theoretically >99% for all size fractions of PM[28]. However, all the facilities investigated in this study showed values lower than such theoretical reference values. We suspect that there were many interfering factors towards the removal efficiency, including the alterations in operating conditions and maintenance of each PM precipitator, types of meats charbroiled, test conditions, etc. In addition, oil mist and vapor generated from fat droplets can be an obstacle that gradually decreases the removal efficiency. They can be accumulated easily on pipelines and walls of precipitators via coagulation and solidification, when oil mist and vapor fly through the precipitators. Furthermore, fat droplets are vaporized or splattered into the smoke aerosol because the liquid grease droplets melt from fat deposits due to heating[29].

Fig. 2 shows the removal efficiency of PM according to particle diameter distribution (31 channels) measured by an OAS. PM <0.4 μm were removed with the least efficiency, while PMs with a diameter <2.5 μm were removed almost completely. In the case of restaurants A, B, and E, the removal efficiency of PM was fairly stable in the overall particle size distribution. However, those of restaurants C and E showed different patterns. The removal efficiency of the latter two was relatively low at PM about <1 μm and >20 μm, respectively.

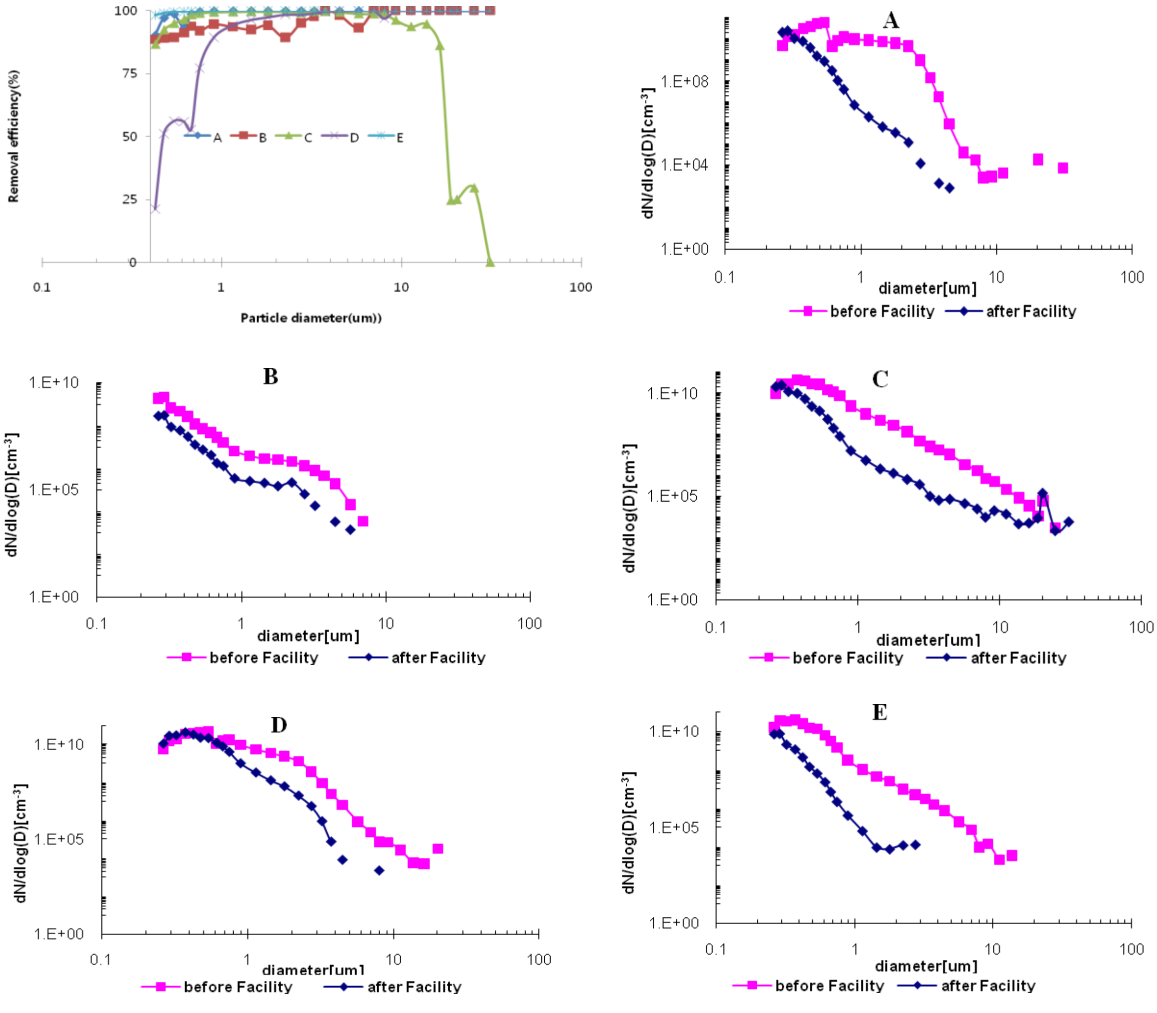

**FIGURE 2.** Removal efficiency of TSP as a function of particle diameter: (A) ESP, (B) ESP, (C) combination (ESP + catalyst), (D) ESP, and (E) BF.

### CONCLUSION

In this research, the concentrations of $PM_{2.5}$, $PM_{10}$, and TSP were measured from five UFC restaurants, and their concentration data were used to derive the removal efficiency between different control facilities. The mean mass concentrations (μg $m^{-3}$) of three PM fractions ($PM_{2.5}/PM_{10}$/TSP) measured from five restaurants were 15,510/15,701/17,175 (C); 8,525/10,760/12,676 (B); 5,168/7,084/7,084 (P1); 16,886/19,413/19,891 (P2); and 22,409/22,412/22,414 (I). A brief glance over the PM data indicates that the concentrations of large intestine tend to peak compared to other meat types. Furthermore, PM released from UFC restaurants in this study was mainly composed of $PM_{2.5}$; 0.73–1.00 ($PM_{2.5}/PM_{10}$) and 0.85–1.00 ($PM_{10}$/TSP). As a result, the control of $PM_{2.5}$ appears to be very important, as its dominant fraction can otherwise be exposed to the customer. Considering the fact that $PM_{2.5}$ was the major component of PM emissions in UFC restaurants, one needs to establish the control strategy of $PM_{2.5}$, as an *a priori* target.

In order to estimate emission rates of $PM_{2.5}$, $PM_{10}$, and TSP, their emission factors were developed for each UFC restaurant investigated in this study. Emission factors of C were the largest in all particle fractions. According to our calculations, the emission rates of $PM_{2.5}$, $PM_{10}$, and TSP were estimated to be 446, 500, and 542 ton $year^{-1}$ in Seoul, respectively. Furthermore, if the contribution of UFC restaurants is extrapolated from our study, it is estimated to comprise 2.4% of $PM_{10}$ emissions from all different sources in Seoul. Removal efficiencies of PM against the major control facilities operated at each restaurant were estimated to be in the range of 54.62–98.98% ($PM_{2.5}$), 54.76–98.98% ($PM_{10}$), and 89.61–99.96% (TSP). In addition, PM with a diameter <10 μm was removed almost completely by the control facilities at most restaurants.

This study was undertaken to describe the basic features of PM emissions from UFC restaurants in Seoul on the basis of PM data measured at each size fraction. The measurement data were used to estimate their emission rates and removal efficiencies against precipitators. The results of our study should be helpful in establishing a database for regional and national emission inventories of PM from UFC restaurants, and to offer appropriate strategies to control air quality in those public facilities.

### ACKNOWLEDGMENTS

This work was supported by a National Research Foundation of Korea (NRF) grant funded by the Ministry of Education, Science and Technology (MEST) (No. 2010-0007876).
